# Hypodermic Injections in the Treatment of Syphilis

**Published:** 1869-08

**Authors:** J. Sherman

**Affiliations:** Lecturer on Orthopœdic Surgery in the Chicago Medical College; 81 Monroe Street


ARTICLE XXX.

### HYPODERMIC INJECTIONS IN THE TREATMENT OF SYPHILIS.

By J. SHERMAN, M.D., Lecturer on Orthopœdic Surgery in the Chicago Medical College.

This mode of introducing remedies in the treatment of syphilis is at present creating considerable interest in the profession. Its application in this disease is not as recent as the reading of some articles would lead one to suppose.

Over three years ago, the injection of calomel and glycerine was practiced with success, as regarded the constitutional effect, but was objectional, from the fact of its frequently producing an abscess at the point of introduction. Some other salts of mercury were also employed, but discarded for the same reason. In the spring of 1868, while in Vienna, I had the opportunity of witnessing the treatment of syphilitic skin diseases, by Prof. HEBRA. He was then using the subdermic injection of hyd. chlor. corr., with marked success, in all secondary lesions. Theoretically, one would suppose this preparation more irritating than any other salt of the metal; practically, it is the reverse, it being the only one suitable for use in this manner.

Within the last six months I have treated quite a number of secondary affections by this method exclusively, and have obtained more prompt results than by administration of remedies through the stomach.

During the early stage of syphilis, *viz.:* the period of the presence of the chancre, digestion is in most cases as active as when the disease is absent. During this time the patient is also more anxious in regard to his condition, and follows more closely the laws of hygeine and the direction of his medical adviser, consequently the anæmia and digestive disturbance is not as marked as in the later manifestations. During this stage remedies by the stomach are well tolerated, and produce results fully as favorable as when the subdermic treatment is used. It is the secondary and later symptoms that yield more promptly to the hypodermic injection.

Then we find our patient often suffering with gastric disturbance, and all the despondency which a return of the disease in another form brings upon those who suppose themselves cured of the malady with the healing of the primary ulcer, the stomach is now needed for the administration of tonics and nutritious food, that the system may gain that strength and tone so necessary for the elimination of any poison. The hypodermic use of mercury has not half the tendency to produce disturbance of the stomach or salivation as when given in the usual method. Its effect is much more rapid, and the inconvenience of administration less to the patient. The amount of hyd. chlor. corr. may vary from one-eighteenth ($\frac{1}{18}$) to one-twelfth ($\frac{1}{12}$) of a grain, dissolved in a drachm of distilled water and injected once every 24 hours. The back, between the scapulæ, will be found the most convenient locality for introducing the syringe.

Here the skin is but little sensitive, and the prominence of the scapulæ protect this point from the contact of the clothing.

A distance of two inches should be selected from the first point for the second operation. The injecting of preparations of iodine have not been found practical, their irritating effects being too great to permit their use.

Special care should be taken to prevent the admission of air at the time of injecting; as it is a frequent cause of suppuration.

*81 Monroe Street.*

